# Supraventricular Tachycardia and Postural Orthostatic Tachycardia Syndrome Overlap: A Retrospective Study

**DOI:** 10.19102/icrm.2021.120201

**Published:** 2021-02-15

**Authors:** Zeid Nesheiwat, Arooge Towheed, Joseph Eid, Jeremy Tomcho, Pinang Shastri, Carson Oostra, Beverly Karabin, Blair Grubb

**Affiliations:** ^1^Department of Internal Medicine, The University of Toledo Medical Center, Toledo, OH, USA; ^2^Department of Cardiac Electrophysiology, The Georgetown University/Medstar Washington Hospital Center, Washington DC, USA; ^3^Division of Cardiovascular Medicine, The University of Toledo Medical Center, Toledo, OH, USA

**Keywords:** Ablation, autoimmune, dysautonomia, electrophysiological study, syncope

## Abstract

Postural orthostatic tachycardia syndrome (POTS) and supraventricular tachycardia (SVT) are disease states with distinctive features but overlapping clinical manifestations. Currently, studies on the presence of underlying SVT in patients with POTS are lacking. This retrospective study analyzed 64 patients [mean age: 43 years; 41 (61%) women] who had a POTS diagnosis and were found to have concomitant SVT during rhythm monitoring from September 1, 2013 to September 30, 2019 at our Syncope and Autonomic Disorders Clinic. The outcomes assessed were changes in disease severity, frequency of symptoms, heart rate, and blood pressure between before and after SVT ablation. The most frequent types of SVT noted on the electrophysiologic study were atrioventricular nodal reentrant tachycardia (57.81%), atrial flutter (29.68%), atrioventricular reentrant tachycardia (9.37%), atrial tachycardia (1.56%), and junctional tachycardia (1.56%). After SVT ablation, all 64 patients experienced an improvement in symptoms. Palpitations and lightheadedness experienced the most improvement after the procedure (72% vs. 31%; p < 0.001 and 63% vs. 22%; p < 0.001, respectively). There was a significant improvement in the resting heart rate (81.1 ± 12.8 vs. 75.8 ± 15.6 bpm; p < 0.002), but the orthostatic tachycardia on standing persisted (93.6 ± 16.5 vs. 77.3 ± 19.8 bpm; p = 0.14). Underlying SVT in patients with POTS can be missed easily. A strong suspicion and long-term ambulatory cardiac rhythm monitoring can help in diagnosing the condition.

## Introduction

Postural orthostatic tachycardia syndrome (POTS) is the most common form of orthostatic intolerance in young people (predominantly premenopausal women), with a prevalence of 0.2%.^1^ Affecting more than 500,000 individuals in the United States alone, its symptoms can be debilitating and can significantly reduce the quality of life.^2^ POTS has often been hypothesized to be a heterogeneous disorder; however, a growing body of evidence suggests that POTS may have an immune-mediated pathogenesis.^3^ The most common symptoms of orthostatic intolerance include syncope, palpitations, fatigue, weakness, sleep disturbances, and exercise intolerance.^4,5^ The typical tachycardia seen in POTS is a sinus tachycardia. Sinus tachycardia is usually a normal physiological response to conditions in which the sympathetic nervous system is activated and the parasympathetic nervous system is inhibited, such as exercise, fever/infection, anemia, anxiety, and pain. Other cardiac causes of sinus tachycardia can be mistaken for POTS and appropriate evaluations should be pursued as guided based on clinical judgment. Inappropriate sinus tachycardia (IST) is sometimes confused with POTS but occurs independently of body position.^6^ In contrast, the tachycardia in POTS is triggered by orthostatic stress, with POTS patients usually presenting a normal resting supine heart rate. As tachycardia is a cardinal manifestation of POTS, an underlying supraventricular tachycardia (SVT) can be easily missed in this patient population. Moreover, the presence of coexisting arrhythmia can make symptoms difficult to control. Assessing and treating POTS patients for underlying SVT can improve their POTS symptoms and, thus, their quality of life.^7^ The purpose of this study was to bring forth the observation that SVT and POTS can occur together. A comprehensive evaluation with an eye for atypical manifestations and subsequent long-term rhythm monitoring of patients can be critical for diagnosis.

## Methods

This retrospective cohort study was performed after obtaining institutional review board approval from the University of Toledo. The need for informed consent was waived due to the retrospective nature of the study. Study participants were identified from among patients referred to our Syncope and Autonomic Disorders Clinic. Patient data were extracted from our electronic medical records system from September 1, 2013, to September 30, 2019. All patients who already had a diagnosis of POTS and were found to have concomitant SVT were included in this study. POTS was defined as a clinical syndrome lasting for at least six months that is characterized by an increase in heart rate of at least 30 bpm within five to 10 minutes of quiet standing or upright tilt, without orthostatic hypotension (> 20-mmHg drop in systolic blood pressure) and which is accompanied by frequent symptoms that occur with standing such as syncope, presyncope, lightheadedness, palpitations, dyspnea, and fatigue.^1^ Patients were diagnosed based on the 2015 Heart Rhythm Society expert consensus statement.^1^ In patients with a high clinical suspicion for POTS, orthostatic vital signs were performed initially after ensuring adequate hydration; if the result was positive, then the diagnosis of POTS was made, while, if the result was negative but the clinical suspicion remained high, a tilt-table test was conducted next to help evaluate vital signs over a prolonged period.

Separately, SVT was defined as any arrhythmia originating above and including the bundle of His with atrial and/or ventricular rates of greater than 100 bpm and explicitly excluding atrial fibrillation. SVT encompassed atrial ventricular nodal reentrant tachycardia (AVNRT), atrioventricular reentrant tachycardia (AVRT) utilizing an accessory pathway, atrial tachycardia (including focal and multifocal), and macroreentrant atrial tachycardia (including typical atrial flutter) for the purposes of our study.^8^ The SVT diagnosis was made by the review of 12-lead electrocardiograms, 48-hour Holter monitoring, 30-day event monitoring, implantable loop recordings, and pacemaker recordings. These diagnostic tests were deployed in patients with atypical POTS symptoms, such as nocturnal symptoms, symptoms appearing while supine, sudden onset and offset of symptoms, and difficult-to-treat POTS. The baseline data included age, sex, weight, height, body mass index, frequency of other comorbidities, and medications. The outcomes (improvement, worsening of symptoms) and orthostatic vitals (heart rate and systolic/diastolic blood pressure, sitting and standing) before and after SVT ablation were assessed.

All patients underwent a standard electrophysiologic study (EPS), during which time, programmed electrical stimulation was conducted inducing SVT that matched their clinical arrhythmias. Although there is a lack of sensitivity and specificity of EPS for certain SVTs, successful radiofrequency ablation (RFA) without sinus-mode modification was conducted within our institution, using activation and voltage mapping in all patients. No complications were reported after any of the procedures.

### Statistical analysis

The Statistical Package for the Social Sciences version 26.0 software program (IBM Corp., Armonk, NY, USA) was used to conduct descriptive statistical analysis. Normally distributed data were described with mean ± standard deviation values, whereas nonparametric data were described with median and range values. A McNemar test was completed to compare symptoms and medications between before and after ablation. The monitoring window used ranged from the time of the outpatient visit right before RFA to three months after RFA. A paired-samples t-test was applied to compare heart rate and blood pressure responses before and after ablation during sitting and standing. A two-tailed p-value of less than 0.05 was considered to be statistically significant.

## Results

A total of 3,550 patients were seen in the Syncope and Autonomic Disorder Clinic from 2013 to 2019, 2,822 (79%) of whom were female. A total of 64 patients [mean age: 43 years; 41 (61%) women] who had a POTS diagnosis were found to have concomitant SVT during rhythm monitoring from September 1, 2013 to September 30, 2019 at our institution. Those patients who had irregular symptoms of POTS or who were difficult to manage (ie, on multiple medications without any improvement of symptoms) were tested for underlying SVT. The most common method of diagnosis was the use of tracings from an implantable loop recorder (n = 19), 48-hour Holter monitor (n = 14), or pacemaker (n = 5). SVT was diagnosed by reviewing Holter monitoring with a 30-second cutoff from onset. Four patients who already had pacemakers implanted prior to the study (two were implanted due to neurocardiogenic syncope, one was received for sick sinus syndrome, and one was placed for intractable atrial fibrillation from cardiac amyloidosis and multiple myeloma). The last pacemaker was implanted two years after SVT RFA for cardiac asystole of an unrelated cause. **Table 1** shows the baseline patient characteristics. **Table 2** shows the medications used before and after RFA. Midodrine usage exhibited the only statistically significant change found (p = 0.016); all 12 patients who were on midodrine pre-RFA were taken off midodrine post-RFA. There were no other significant changes in medication usage throughout the study.

The most frequent subtype of SVT noted on the EPS was AVNRT (57.81%), followed by atrial flutter (29.68%), AVRT (9.37%), focal atrial tachycardia (1.56%), and junctional tachycardia (1.56%) **(Table 3)**.

**Figure 1:**
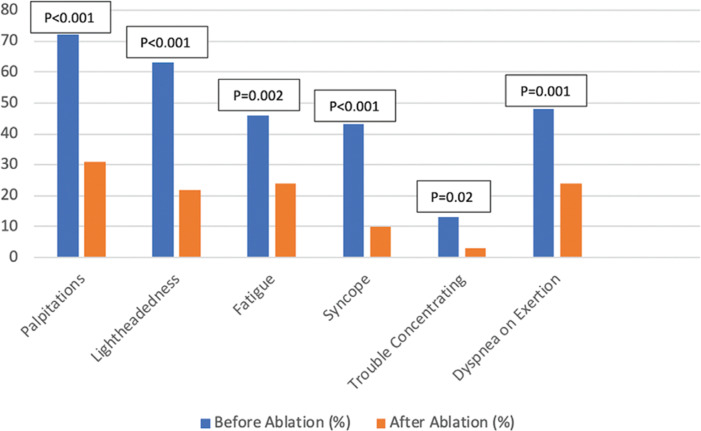
Percentages of symptoms reported by patients before and after ablation, respectively.

Following ablation, there was a statistically significant decline in the prevalence of symptoms reported before ablation. Palpitations and lightheadedness showed the most significant improvements after ablation (72% versus 31%; p < 0.001 and 63% versus 22%; p < 0.001, respectively) **(Table 4 and Figure 1)**. No significant change was made in patients’ therapeutic regimens between before and after ablation to avoid confounding of results.

There was a statistically significant decrease in the sitting heart rate following ablation (81.1 ± 12.8 bpm versus 75.8 ± 15.6 bpm; p < 0.002), but the orthostatic tachycardia persisted. There was no substantial change in the standing heart rate. A comparison of vital signs between before and after ablation is presented in **Table 5**.

Pacemakers and loop recorders continued to be evaluated following ablation. There was no recurrence of SVT during follow-up within three months of RFA. No regular SVT follow-up was conducted after three months unless there was a clinical indication and/or a return or worsening of symptoms. POTS was easier to manage after RFA on the basis of symptomology control.

## Discussion

In this retrospective cohort study, we examined the presence of hidden SVT in patients with POTS. Although there are reports of IST and POTS overlap, there is no description of underlying SVT in POTS.^9^ SVT can be easily overlooked in POTS patients as both are disease states with distinctive features but similar clinical manifestations.^1^ Clinicians must be very astute and should consider alternative diagnoses in patients whose symptoms do not fully fit POTS and/or are difficult to treat. Nocturnal symptoms, symptoms that appear when lying flat and sudden onset and offset of symptoms not related to the position should serve as warnings for physicians.^6^ A low threshold for ambulatory long-term rhythm monitoring with a 48-hour Holter monitor, 30-day event recorder, or implantable loop recorder should be maintained. Multiple studies have proven the efficacy of long-term rhythm monitoring to diagnose occult/hidden arrhythmias, which would otherwise rarely be detected.^10–12^ This is because the symptomology between SVT and POTS can be quite similar.

Treating the underlying rhythm disorder will greatly help in symptom relief and make it easier to manage POTS and potentially limit the medications used for symptomatic care as seen by the decreased use of midodrine in our population. Ablation has become the standard of care for most patients with SVT.^8,13^ In our cohort, all patients underwent successful RFA with no recurrence of arrhythmias. Although this did not wholly resolve their orthostatic tachycardia, it significantly alleviated their symptoms **(Figure 1)**.

The etiology of SVT in POTS patients is unclear. A significant subset of POTS patients experience persistent sympathetic activation due to hemodynamic changes associated with POTS.^14^ It has been shown that persistent adrenergic stimulation can lead to the activation of existing potential reentry circuits, enhanced automaticity, and early or delayed after-depolarization–associated triggered activity.^15,16^ We believe that this might be the reason for the development of SVT in these patients, thereby causing further aggravation of their symptoms. In addition, the prevalence of SVT in our patient population was 1.8%, which is eight times higher than that of the general population.^17^ Further research such as subgroup analysis or a larger cohort trial is needed to help establish the true prevalence of SVT in POTS.

### Limitations

This study has several limitations that should be acknowledged. First, it was a retrospective, observational research study and therefore has all of the limitations of this study type such as selection bias. The small sample size constitutes another limitation. Moreover, confounding could not totally be accounted for because of the study’s retrospective nature and the inability to fully control medication changes during the study period. Also, the duration of SVT was not recorded and the study was conducted in a single center. Despite these limitations, the findings of the study substantiate the utility of long-term rhythm monitoring in these patients.

## Conclusion

In this observational study, patients diagnosed with POTS had concomitant SVT. The presence of SVT leads to atypical manifestations and difficulty in managing symptoms. Maintaining a low threshold for long-term rhythm monitoring in such patients can help diagnose these arrhythmias. Ablation can help cure SVT and thereby significantly improve the quality of life of patients.

## Figures and Tables

**Table 1:** Baseline Characteristics of the Study Participants (n = 67)

Age, mean ± SD	47.4 ± 18.8 years
Female sex, n (%)	41 (61%)
Weight, mean ± SD	85.3 ± 21.2 lbs
Height at commencement, median	1.7 m (0.5 m)
BMI, mean ± SD	29.4 ± 6.5 kg/m^2^
Tobacco use, n (%)	11 (16%)
Alcohol use, n (%)	12 (18%)
Congenital heart disease, n (%)	2 (3%)
Congestive heart failure, n (%)	10 (15%)
COPD/ILD, n (%)	2 (3%)
Diabetes, n (%)	7 (10%)
Pulmonary hypertension, n (%)	3 (4%)
Thyroid disease, n (%)	6 (9%)
Hypertension, n (%)	28 (42%)
ECG characteristics, mean ± SD	
P-wave duration	0.05 ± 0.02 s
P–R interval	152.5 ± 28.9 ms
QRS	101 ± 30.7 ms
Echocardiographic characteristics, mean ± SD	
LV ejection fraction	53.4% ± 13.3%
LAVI	26.6 ± 12.1 mL/m^2^
RAA	16.1 ± 7.6 cm^2^

BMI: body mass index; COPD: chronic obstructive pulmonary disease; ECG: electrocardiogram; ILD: interstitial lung disease; LV: left ventricle; LAVI: left atrial volume index; RAA: right atrial area; SD: standard deviation.

**Table 2:** Medications Used Before and After Ablation

Medication	Before Ablation	After Ablation	p-value
β-blocker	56.7%	50.7%	0.359
Ivabradine	6.0%	3.0%	0.625
Gabapentin/pregabalin	6.0%	9.0%	0.625
Midodrine	12%	0%	0.016
Epogen	1.5%	0%	1.00
SSRI/SNRI	29.9%	28.4%	1.00
Pyridostigmine	7.5%	3.0%	0.625
Desmopressin	1.5%	4.5%	0.50
Fludrocortisone	6.0%	0%	0.25
Sympatholytic	6.0%	3.0%	1.00
IVS	1.5%	1.5%	1.00
Amphetamine	7.5%	4.5%	0.50

IVS: intravenous saline; POTS: postural orthostatic tachycardia syndrome; SVT: supraventricular tachycardia; SSRI: selective serotonin reuptake inhibitor; SNRI: selective norepinephrine reuptake inhibitor.

**Table 3:** Frequency of Different Types of SVT in Patients with POTS

Arrhythmia	n (%)
AVNRT	37 (57.81%)
Atrial flutter	19 (29.68%)
Atrial tachycardia	01 (1.56%)
AVRT	6 (9.37%)
Junctional tachycardia	01 (1.56%)

AVNRT: atrioventricular nodal reentrant tachycardia; AVRT: atrioventricular tachycardia; POTS: postural orthostatic tachycardia syndrome; SVT: supraventricular tachycardia.

**Table 4:** Most Commonly Reported Symptoms Before and After Ablation

Symptom	Before, n (%)	After, n (%)	p-value
Palpitations	46 (72%)	20 (31%)	< 0.001
Lightheadedness	40 (63%)	14 (22%)	< 0.001
Fatigue	30 (46%)	15 (24%)	0.002
Syncope	28 (43%)	6 (10%)	< 0.001
Trouble concentrating	8 (13%)	2 (3%)	0.02
Dyspnea on exertion	31 (48%)	15 (24%)	0.001

**Table 5:** Vital Signs of Patients Before and After Ablation

	Before	After	p-value
Sitting HR, mean ± SD	81.1 ± 12.8 bpm	75.8 ± 15.6 bpm	0.002
Standing HR, mean ± SD	93.6 ± 16.5 bpm	77.3 ± 19.8 bpm	0.144
Sitting SBP, mean ± SD	125.6 ± 14.1 mmHg	122.9 ± 18.4 mmHg	0.001
Sitting DBP, mean ± SD	74.4 ± 10.4 mmHg	72.5 ± 12.8 mmHg	0.017
Standing SBP, mean ± SD	117.6 ± 15.1 mmHg	116.4 ± 13.3 mmHg	0.62
Standing DBP, mean ± SD	73.2 ± 10.8 mmHg	70.6 ± 11.5 mmHg	0.358

DBP: diastolic blood pressure; SBP: systolic blood pressure; SD: standard deviation.
